# Biliary Atresia Associated with Polysplenia Syndrome, Situs Inversus Abdominus, and Reverse Rotation of Intestine

**Published:** 2012-06-01

**Authors:** Bilal Mirza, Shahid Iqbal, Afzal Sheikh

**Affiliations:** Department of Pediatric Surgery, The Children’s Hospital and the Institute of Child Health Lahore, Pakistan

**Dear Sir**

In 25% of cases of biliary atresia associated malformations are present; polysplenia constitute 10-50% of these associated anomalies. Biliary atresia found associated with polysplenia syndrome, heterotaxy, and reverse rotation of intestine, in isolation or in various combinations; however its association with polysplenia syndrome, situs inversus abdominus, and reverse rotation of gut in the same patient is not reported in English language literature [1,2].

A 2-month-old female infant presented with jaundice, clay colored stools, and abdominal distension since the first week of life. She was a product of consanguineous marriage and born via normal vaginal delivery. General physical examination revealed a vitally stable infant with obvious jaundice and abdominal distension. Abdominal examination revealed hepatomegaly. Her laboratory investigations showed conjugated hyperbilirubinemia (total bilirubin 14 mg/dl, and direct bilirubin 8 mg/dl). Gallbladder was not visualized on ultrasound of the abdomen. On HIDA scan no excretion of the radiopharmaceutical tracer was noted. At operation the gallbladder was found atretic (Fig. 1); per-operative cholangiogram confirmed extrahepatic biliary atresia. The liver was enlarged and central in position. Stomach and two big spleens (non-floating) were present on the right side of the abdominal cavity (Fig. 1). The duodenum was entirely intra-peritoneal and transverse colon was retroperitoneal in position (Fig. 2).

**Figure:**
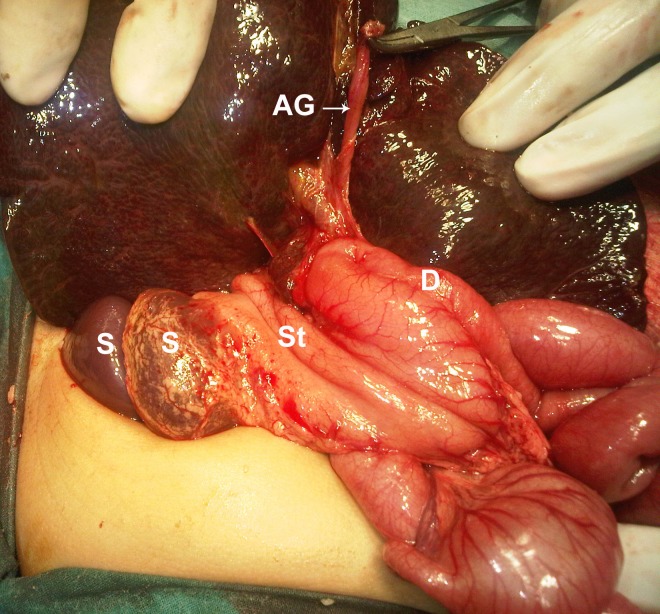
Figure 1: Showing atretic gallbladder (AG); stomach (St) and two spleens (S) on right side; and intraperitoneal duodenum (D).

**Figure:**
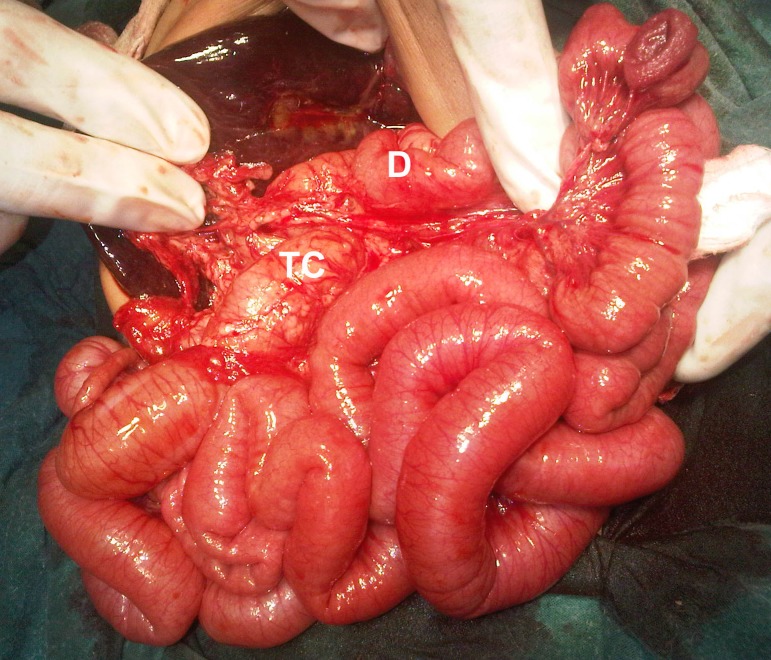
Figure 2: Showing intraperitoneal duodenum (D) and retroperitoneal transverse colon (TC).

The atretic gallbladder and portal plate were dissected meticulously. Roux-en Y hepatico-jejunostomy was performed without going through the transverse colon window. Both the spleens were left as such. No effort was made to correct reverse rotation. The postoperative recovery was uneventful. Patient was allowed oral feeding on 5th day of operation and discharged on 7th day. Liver biopsy showed early cirrhotic changes. Postoperatively, echocardiography and chest x-ray did not show intrathoracic heterotaxy. She was on follow up of gastroenterology department for further management.

Polysplenia syndrome is associated with a number of anomalies i.e. heterotaxy of abdominal or thoracic organs, malrotation of gut, biliary atresia, reverse rotation of gut, intestinal atresia, retroperitoneal teratoma, vena caval anomalies, cardiac, and lung anomalies. Few case reports described the association of biliary atresia with polysplenia syndrome, situs inversus, and immotile cilia syndrome. Polysplenia syndrome is more frequently associated with vascular and cardio-pulmonary anomalies however the work of Chandra proved that a subclass of patients of polysplenia syndrome had biliary atresia that were associated with gastrointestinal and respiratory anomalies [1-3].

The abdominal heterotaxy in a case of biliary atresia has few surgical implications. These problems are related to the orientation of roux-en-y loop. In a usual case of biliary atresia, the roux-en-y loop is passed through a rent made in the mesentery of transverse colon, however in case of malrotation and reverse rotation of gut the there could be difficulties in the orientation of the roux-en-y loop. This difficulty is further incremented by mirrored anatomy of abdominal viscera. In our case the stomach and spleens were lying on right side of the abdomen. The duodenum was intraperitoneal and transverse colon retroperitoneal, therefore mesentery of transverse colon was not available. Thus we have to take the roux-en-y loop directly to the portal plate for hepatico-jejunostomy. To conclude, biliary atresia polysplenia syndrome is a rare occurrence. The operating surgeon must be aware of the surgical implications that may occur in case of associated malrotation/reverse rotation, and mirrored alimentary tract anatomy. .

## Footnotes

**Source of Support:** Nil

**Conflict of Interest:** None declared
